# Single-Port Laparoscopic Assisted Transcrotal Orchidopexy for Palpable Inguinal Canalicular Cryptorchidism Accompany With Indirect Inguinal Hernia

**DOI:** 10.3389/fped.2018.00293

**Published:** 2018-10-09

**Authors:** Yazhen Ma, Jianhui Cai, Suolin Li, Wenbo Wang, Lin Liu

**Affiliations:** ^1^Department of Surgery, Graduate School of Hebei Medical University, Shijiazhuang, China; ^2^Department of Pediatric Surgery, The Second Hospital of Hebei Medical University, Shijiazhuang, China; ^3^Department of General Surgery, Hebei General Hospital, Shijiazhuang, China

**Keywords:** cryptorchidism, palpable undescended testis, indirect inguinal hernia, laparoscopy, orchidopexy, treatment

## Abstract

**Purpose:** To assess the outcomes of a novel laparoscopic assisted transcrotal orchidopexy (LATO) combined with percutaneous extraperitoneal closure (PEC) for palpable inguinal canalicular cryptorchidism accompany with indirect inguinal hernia, and evaluate its safety and efficiency.

**Materials and Methods:** A retrospective cohort study for single-port LATO-PEC and traditional inguinal orchidopexy (TIO) was performed between 2011 and 2014. Totally 53 children with both palpable inguinal canalicular testes and indirect inguinal hernia were included. Median patient age was 15month (range, 6 months to 4 years). Of them, 35 patients underwent LATO-PEC procedure, utilizing an umbilical trocar for laparoscope, transcrotal dissection for orchidopexy, and an inner two-hooked cannula for ligation of the patent processus at the level of the internal ring. Three of them were bilateral, 12 on the left side and 20 on the right. Eighteen patients received TIO, seven of them on the left side and 11 on the right. Patient demographics, surgical technique, complications, and clinical outcomes were reviewed. Follow-up visits were performed to reassess position and size of the testes.

**Results:** All 56 undescended testes were delivered into the scrotum successfully. In the LATO-PEC group, nine contralateral herniorrhaphy were accomplished simultaneously. Fifteen contralateral patent processus vaginalis (PPVs) in 32 unilateral undescended testis (UDT) were newly confirmed during the laparoscopy, while 6 of them received percutaneous extra-peritoneal herniorrhaphy for visible inguinal bubble in pneumoperitoneum condition. No additional port placement or conversion to open procedure was needed. Mean operative time for unilateral and bilateral LATO-PEC in this study was (37.81 ± 5.23) min and (53.33 ± 2.98) min, respectively. In TIO group, mean operative time was (41.11 ± 8.67) min. There was no statistical difference in operative time between the two approaches for unilateral UDTs (*p* = 0.098). Median follow-up interval was 24 months (range, 12–84 months). No operative complications were found in either group to date.

**Conclusions:** Singe-port LATO-PEC is a safe, effective, and cosmetic choice for inguinal canalicular cryptorchidism accompany with indirect inguinal hernia, minimizing injuries to the vas deferens and testicular vessels. Laparoscopy can provide a diagnostic and therapeutic solution of contralateral PPV.

## Introduction

Cryptorchidism, also called UDT, is one of the commonest genital malformation in boys. Patients are best diagnosed clinically, and treated by surgical orchidopexy (1). The majority of UDT are palpable. Bilateral presentation is found in 10–20% of cases (2, 3). Although the etiology is unknown, cryptorchidism is usually accompanied by PPVor hernia (4, 5).

The management for patients with both clinical hernia and UDT includes testis mobilization and hernia closure. Traditionally, we choose a transverse incision in the inguinal area and a scrotal incision to apply orchidopexy and herniorrhaphy simultaneously. Since transcrotal orchidopexy (TSO) has been reported by Gordon et al. (6), use of the standard Bianchi procedure allows for the vast majority of palpable undescended testicles to be brought comfortably in the scrotum with concomitant trans scrotal ligation of the hernia sac. An additional inguinal incision is used if more proximal dissection is needed for mobilization than a scrotal incision allows (7). When contralateral hernia is found, another inguinal incision is needed.

As the technique of laparoscopic orchidopexy (LO) developed, LO and herniorrhaphy can be proceeded simultaneously with 3 to 4 trocars (8, 9). In 2014, Li et al. reported laparoscopic percutaneous extra-peritoneal closure for pediatric inguinal hernia, using an innovative two-hooked needle (10). Herein, we introduce this laparoscopic assisted transcrotal orchidopexy (LATO) combined with percutaneous extra-peritoneal closure (PEC), for the management of patients with both UDT and clinical hernia, using the inner two-hooked cannula, with obviation of additional working port and groin incision.

## Materials and methods

A retrospective cohort study, from 2011 to 2014, for all LATO-PEC and traditional inguinal orchidopexy (TIO) procedures was performed. Palpable testes near the scrotum who had received single transcrotal orchidopexy were excluded. Patients who had already received a failed trans-inguinal orchidopexy before and patients with non-palpable UDT were all excluded. Patients with abdominal surgery histories before were excluded too. Totally 53 children with palpable inguinal canalicular testes and indirect inguinal hernia were included. Patient age ranged from 6 months to 4 years (mean 15 months).Thirty-five patients underwent LATO-PEC procedure, three of them were bilateral UDT with right inguinal hernia, 12 on the left side and 20 on the right. Contralateral hernia coexist with unilateral UDT in 9. Eighteen patients received TIO, seven of them on the left side and 11 on the right. No patient with bilateral UDT or contralateral hernia was included in TIO group. Patient demographics, surgical technique, complications, and clinical outcomes were reviewed. This study was approved by the joint ethical committee of the Second Hospital of Hebei Medical University (No. 2011L-8). The informed consent was obtained for diagnostic laparoscopy and orchidopexy.

### Surgical technique for LATO-PEC

**Figure 1:**
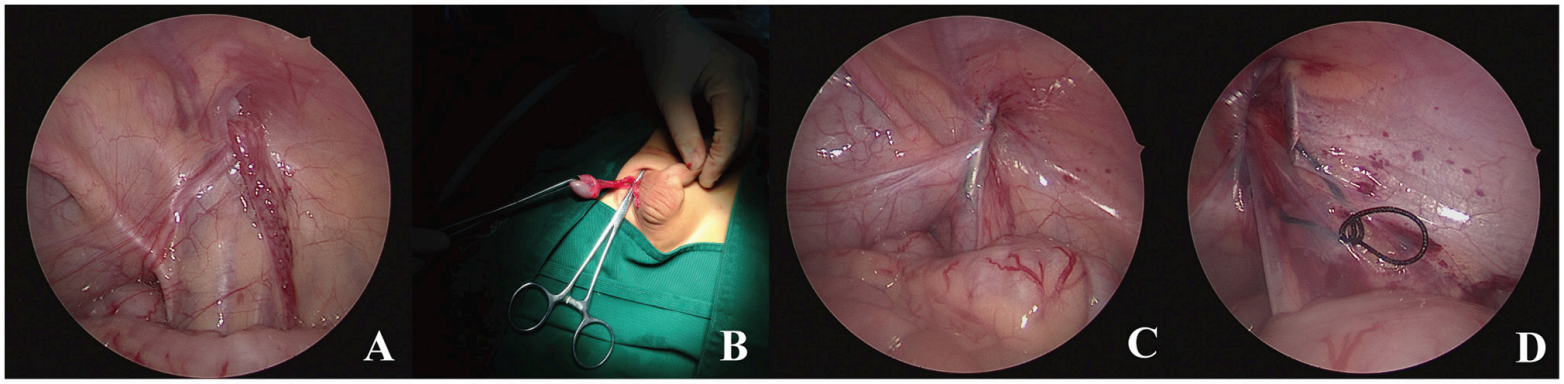
The technique of LATO-PEC. **(A)** The undescended testis in right inguinal canal with hernia. **(B)** Testis pulled out with adequate trans-scrotal mobilization before re-location. **(C)** Suture bringed into abdomen along pre-peritoneal space. **(D)** Internal ring before fixation.

After induction of general anesthesia, the patient was placed in the supine position. A 5-mm umbilical incision was made, followed by the insertion of a 5-mm trocar using an open technique. A 5-mm 30 degree lens was introduced, and pneumoperitoneum was achieved to 8 mmHg, confirming the location and development of affected testes and spermatic cords (Figure 1A), as well as the contralateral situation. When testis was identified lying in the indirect inguinal hernia sac, LATO-PEC procedure was performed.

A transverse incision (8–10 mm length) was then made along the middle scrotum, followed by creation of adequate dartos pouch for re-location. The dartos was cut open and the end of processus vaginalis could be found around the external ring. Because of the ipsilateral hernia, the bottom of hernia sac was much easier to be found and separated from the cord structures. The dartos was further dissected to create a potential “tunnel” into inguinal canal, clearing the lateral tissues around hernia sac along outer front side of peritoneum by means of the laparoscopic guide. The hernia sac was then cut open, so that a clamp could be introduced into the inguinal canal or abdominal cavity through external ring. When testis gubernaculum was clamped and pulled through extra-corporeally, the clamp could further dissect the processus vaginalis off of the spermatic cord for mobility (Figure 1B). When adequate testicular mobilization was achieved, no more transrotal separation of the cremaster muscle and retroperitoneal funiculolysis for proximal ligation of hernia sac would be performed. The descended testis was re-located into scrotal pouch using 4-0 absorbable suture.

After that, laparoscopic percutaneous extra-peritoneal herniorrhaphy was applied, with an inner two-hooked cannula (10). Briefly, the middle of a folding suture (2-0 silk) was placed in distal slot of needle core and inserted into pre-peritoneal space. The suture-loaded cannula further advanced along the medial side of internal orifice of unclosed internal ring, dissecting the Vas Deferens and separating the gonadal vessels into intra-abdominal space (Figure 1C), and release the folding suture in peritoneal cavity. The cannula was gently withdrawn until its tip was within the pre-peritoneal space and re-introduced along the lateral side of internal ring, at the same peritoneal puncture point. The initiated suture was placed in proximal slot and pulled out, then the inner ring was ligated outside (Figure 1D). When contralateral PPV with visible inguinal bubble was determined in pneumoperitoneum, contralateral extra-peritoneal herniorrhaphy could be easily performed by the same cannula simultaneously. Finally the scrotal and umbilical incisions were closed in standard fashion.

### Surgical technique for TIO

A transverse inguinal incision is used (20–40 mm in length). The front side of inguinal canal was cut open first for testis location. Followings are the division of the gubernacular attachment and separation of the cremaster off of the spermatic cord. Then the hernia sac was isolated and ligated in level of the internal ring. Finally the testis was mobilize into the scrotum within a subcutaneous or sub-dartos pouch (11).

Follow-up visits were performed months after surgery to reassess position and size of the testes. Successful orchidopexy was defined as scrotal position of the testis without atrophy or hernia by clinical examination at the time of the follow-up visit.

### Statistical analysis

Data analysis was performed using SPSS 18.0 (SPSS Inc., Chicago, IL, USA). Descriptive characteristics are presented as the means (±SD). Age and follow-up time were assessed with non-parametric test, and all values were expressed as median values with range. Student's *t*-test was used to compare the operative time between unilateral UDT patients received LATO-PEC and TIO. A value of *P* < 0.05 denoted statistical significance.

## Results

All 56 undescended testes of 53 patients were delivered into the scrotum successfully.

In LATO-PEC group, nine contralateral herniorrhaphy were applied simultaneously. Fifteen contralateral PPV in 32 unilateral UDT was newly confirmed by laparoscopy, while 6 of them received PEC for visible inguinal bubble in pneumoperitoneum condition. All three bilateral UDT with right inguinal hernia were found with left PPV, who had received PEC of internal ring. No additional port placement or conversion to open procedure was needed. Incisions was located at umbilicus for lens, scrotum for testis delivering and abdomen for PEC. Mean operating time for unilateral and bilateral LATO in our series was (37.81 ± 5.23) min and (53.33 ± 2.98) min, respectively.

In TIO group, the mean operative time for unilateral UDT was (41.11 ± 8.67) min. There was no statistical difference in operative time between the two approaches for unilateral UDTs (*p* = 0.098).

No intra-operative complications were found in our study and all patients discharged within 2 days after operation. Median follow-up time was 24 months (range, 12–84 months). No patient was lost to follow-up. Ultrasound examination and/or telephone inquiring indicated normal size and location of descended testis during follow-up periods. Wound infection, inguinal hernia, hydrocele, suture granuloma formation, fever, re-ascending, and atrophy were not found in further consultation in out-patient department. The satisfactory cosmetic could be seen in scrotum and inguinal area in LATO-PEC group, as no scar is visible.

## Discussion

Cryptorchidism is one of the most frequently performed surgical problems in children. About 80 percent of them are palpable (2). The palpable undescended testes are often accompanied by hernia or PPV. In 2012, Aggarwal et al. reported that one third of patients with a unilateral palpable undescended testis have a contralateral patent processus (4). Over time, the gold standard for palpable cryptorchidism with inguinal hernia is still inguinal orchidopexy and herniorrhaphy. Palpable undescended testes can easily be repaired either by the traditional 2 incision approach or even a single incision subinguinal approach.

Incision in traditional inguinal approach is only 2 cm in an experienced surgeon's hands. While inguinal canal has to be cut open to achieved adequate dissection and ligation of PPV. Damage to anatomical structure of inguinal canal is inevitable, as well as scar left. Sometimes the scar maybe grow longer and wider as the body grows up, especially in scar physique. Surgical history could be easily found by patient himself, with psychological influence when grow older. Sometimes when hernia is on the opposite side, another groin incision should be necessitaitis.

Single TSO and LO have been reported sparsely, as minimal invasive surgeries for cryptorchidism with avoidance of the groin incisions and better cosmetic results, but followed by limited indications (6, 12, 13).

TSO was first introduced by Bianchi and colleagues and quickly gained wide acceptance for palpable cryptorchid testes (6). Many authors have published their cumulative experience about TSO techniques for palpable UDT management (7, 14). Use of the standard TSO allows for the vast majority of palpable undescended testicles to be brought comfortably in the scrotum with concomitant trans scrotal ligation of the hernia sac. If the testis is palpable near the scrotum, or it can easily be brought down to a dependent scrotal position, a single scrotal approach can be used. However when the testis is in a higher position, such as in the inguinal canal or just at the external ring, TSO brings troublesome complications include injury to the vas deferens and testicular vessels. In 2016, Lopes et al. published articles about post-operation complications (15, 16). Hernia, hydrocele, testicle ascending and atrophy happens, owing to insufficient (retroperitoneal) mobilization of vas and vessels and/or inadequate high ligation of patent processus vaginalis. Another inguinal incision is used if more proximal dissection is needed for mobilization than a scrotal incision allows. Additionally, transcrotal procedure cannot achieve bilateral investigations.

Many authors have published their cumulative experience about LO for palpable and non-palpable UDT (17–19). LO has obvious superiorities such as sufficient retroperitoneal dissection, sufficient testes mobilization and bilateral investigation, as well as easy fixation of the unclosed internal ring. Traditional LO needs 3 trocars on abdomen. One for the laparoscope and 2 for the instruments. An optional 5 or 10 mm scrotal port may be placed for delivering the testis into the scrotal sac if necessary. More trocars cost much and bring more injuries with complications such as omental hernia. Retroperitoneal dissection is not necessary in majority of palpable conditions. More scars are left with bad cosmesis too. In 2011, Sultan et al. reported the single-site laparoendoscopic orchidopexy requiring an single incision (2 cm) around the umbilicus (20). Expensive laparo-angle bending instruments are always needed and inconvenient clashing between camera and instruments prolonged the operation time.

During LO procedure, most surgeons only resect the membranes around internal ring instead of ligation (21, 22). We take it as mandatory to prevent post-operative hernia when clinical hernia is already confirmed. In 2010, Chang reported single-port laparoscopic surgery of inguinal hernia in infants and children (23). Various kinds of single-port laparoscopic herniorrhaphy have been reported and obtained good results. Li et al. have reported a single-port laparoscopic percutaneous extra-peritoneal closure in 2014, using an innovative apparatus for pediatric inguinal hernia and assumed lots of experiences with a two-hooked needle (10). Since then, the apparatus has became a convenient equipment for laparoscopic dissection and ligation around the internal ring.

Longing for more convenience and further minimize the potential invasions to inguinal vessels and abdominal wall, we modified the LATO procedure, a combined Bianchi procedure, with laparoscopic inner ring ligation, only utilizing an umbilical trocar for laparoscope view, transcrotal dissection for orchidopexy, and needle ligation for PEC at the level of the internal ring.

In patients with both palpable UDT and hernia, the inguinal canal and the internal ring are widely opened. Herniorraphy are necessary to avoid post-operation hernia or hydrocele. Single TSO achieves herniorraphy around the superficial ring rather than the inner ring, which can easily result in recurrence of hernia or hydrocele in PPV condition. TIO is known as the most commonly used approach for UDT. Traditional inguinal approach achieves adequate mobilization and high ligation of hernia sac easily, with dissection of inguinal canal muscles. More injuries are inevitable. The LATO-PEC procedure provides a good solution of the contradictions. The laparoscope, sited at umbilicus, can enter into the bottom of hernia sac through the widely opened internal ring and inguinal canal. When the bottom of the hernia sac is cut open, the clamp could be easily introduced into the inguinal canal or abdominal cavity through external ring. The testis can be clamped and pulled through extra-corporeally by means of the laparoscope guide for further mobilization. We manipulate palpable inguinal canalicular cryptorchidism accompany with hernia under the laparoscope guidance, so that the mobilization of testis cord is usually easier and transcrotal oxchidopexy is enough for most testes. When testicles have already been delivered into the scrotum comfortably, PEC can be performed instead of proximal canalicular dissection for ligation of hernia, avoiding possible injuries to the vas deferens and testicular vessels. While additional retroperitoneal hydro dissection was applied sometimes around the internal ring for adequate mobilization if needed. Muscle injuries are minimized. Mobilization difficulties are decreased. Operation time is similar. LATO-PEC procedure can detect the whole pelvic cavity and deal with bilateral anomalies easily. Incidence of contralateral PPV in unilateral and bilateral canalicular UDT is 46.88% (15/32) and 100% (3/3), respectively. Contralateral herniorrhaphy will be easily applied simultaneously when contralateral PPV with inguinal bubble is found in pneumoperitoneum condition. No patient with bilateral UDT or contralateral hernia were included in our TIO group. While the number of bilateral UDT and contralateral hernia in LATO-PEC group is 3 and 9, respectively. Bilateral canalicular UDT seems to be one of the indications for laparoscopy, as well as the unilateral UDT with contralateral anomalies.

No intra-operative complications is noted in our LATO-PEC series of patients. Excessive transcrotal canalicular dissection of spermatic cord is avoided. No visceral or vascular injury occurs. No more port or inguinal incision is needed. The innovated procedure is as safe as the TIO group.

**Figure 2:**
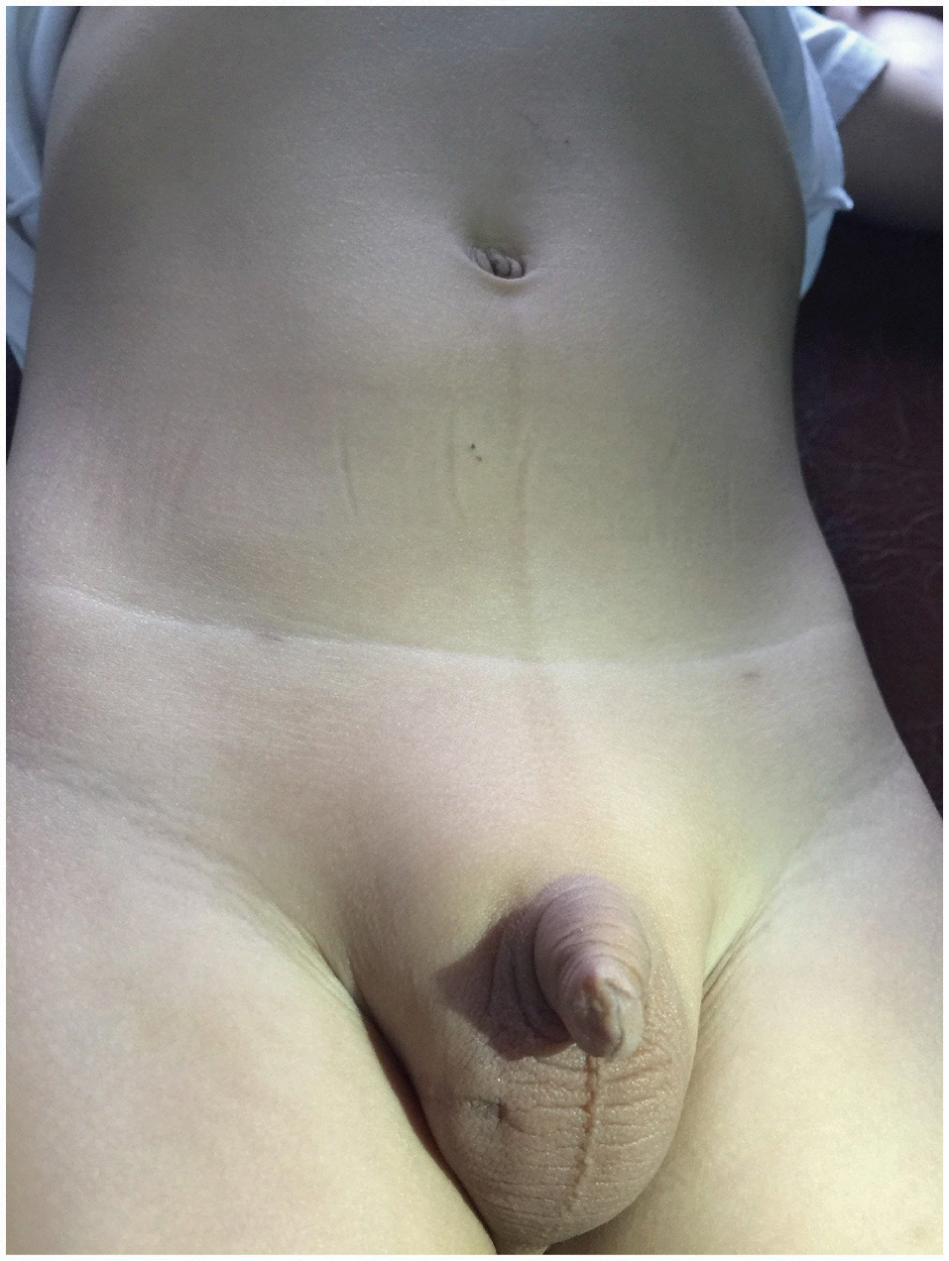
Post-operation appearance of incisions at umbilicus for lens, scrotum for testis delivering, and abdomen for PEC.

Mean operative time for unilateral and bilateral LATO-PEC in our study is (37.81 ± 5.23) and (53.33 ± 2.98) min, respectively. Most transcrotal orchidopexies are performed in 20–30 min (6). Riquelme et al. have reported an operative time of 50 min for LO (24). LATO-PEC is comparable to their series. The absence of laparoscopic equipments do not increase operative times. Scars can be very well concealed along the skin fold of umbilicus and the scrotum (Figure 2). No visible scar is found. LATO-PEC procedure obviates the need for a second trocar. Totally 70 trocars are saved. Savings and cosmetics will be easily achieved.

## Conclusions

Singe-port LATO-PEC procedure is a safe, effective, and cosmetic choice for palpable inguinal canalicular cryptorchidism accompany with indirect inguinal hernia, minimizing injuries to the vas deferens and testicular vessels. Routine inspection of the contralateral internal ring should be performed during laparoscopic orchidopexy. Laparoscopy would provide a diagnostic and therapeutic solution of contralateral hernia. Further studies are warranted to compare the morbidity, ergonomics, cosmesis, and reproducibility of outcomes for various options of minimally invasive surgery for cryptorchidism.

## Author contributions

YM: protocol, project development, data collection, and manuscript writing; JC: protocol, editing, and revising; SL: project development, patient management, and surgery; WW: collection and manuscript writing; LL: collection and manuscript writing.

### Conflict of interest statement

The authors declare that the research was conducted in the absence of any commercial or financial relationships that could be construed as a potential conflict of interest.

## Abbreviations

LATO: laparoscopic assisted transcrotal orchidopexy
PEC: percutaneous extraperitoneal closure
TIO: traditional inguinal orchidopexy
PPV: patent processus vaginalis
UDT: undescended testis
TSO: transcrotal orchidopexy
LO: laparoscopic orchidopexy.
